# The Glucose-Lowering Effects of Coconut Oil: A Case Report and Review of the Literature

**DOI:** 10.1155/2020/8841781

**Published:** 2020-12-27

**Authors:** Samar Malaeb, Christopher Spoke

**Affiliations:** ^1^Division of Diabetes, Endocrinology and Metabolism, Department of Medicine, University of Minnesota, Minneapolis, MN 55407, USA; ^2^Department of Medical Education, Abbott Northwestern Hospital, Minneapolis, MN 55455, USA

## Abstract

**Background:**

Coconut oil, a saturated fat comprised mostly of the medium-chain fatty acid, lauric acid, has become increasingly popular over the past few decades due to its touted anti-inflammatory, metabolic, and lipid-lowering properties. There have been many studies with mixed results evaluating the effects of coconut oil consumption on lipid metabolism and cardiometabolic risk. However, the effects on glucose metabolism are less clear. There are few trials on the effects of coconut oil on glucose homeostasis but no case reports prior to the current one.

**Case:**

We present a case of a 66-year-old man with a history of type 2 diabetes managed with insulin who developed recurrent hypoglycemia and required reduction in insulin therapy quickly after consuming coconut oil supplementation.

**Conclusion:**

This is the first known case report of coconut oil supplementation in a diabetic patient on insulin resulting in hypoglycemia. Review of the literature shows that coconut oil supplementation can have a favorable effect on glycemic control, possibly through phenolic compounds mediating anti-inflammatory effects. This effect is inconsistent throughout the studies reviewed, likely due to variations in types of coconut oil supplementation and scarcity of trials. Further research is required both in animal models and in humans before coconut oil intake is widely advised and popularized. This is especially true in patients with diabetes, who are at increased risk of cardiovascular disease, and in whom reduction in saturated fat intake is advised.

## 1. Background

The use of coconut oil has become increasingly popular over the past few decades due to claims regarding anti-inflammatory, metabolic, and lipid-altering properties. As a result, there has been an increase in demand for research and clinical trials to evaluate the cardiometabolic and energy metabolism effects of coconut oil. The usual coconut oil commercial product is obtained from the fresh mature kernel of the coconut plant as either refined, bleached, and deodorized coconut oil or, more recently, virgin (i.e., unrefined) coconut oil [1, 2]. The predominant type of fat in coconut oil is lauric acid (comprising about 50%), with smaller amounts of myristic and palmitic acid. Lauric acid is a saturated fatty acid (SFA) with a structure that is C12 : 0, classifying it as a medium-chain fatty acid (MCFA) [1]. While recent dietary guidelines have recommended a diet low in SFA, specifically aiming for less than 10% of daily total caloric intake and replacing SFA with unsaturated fats [3, 4], coconut oil's medium-chain fatty acid property, as well as its high content of polyphenolic compounds, which have been implicated in its anti-inflammatory and enhanced metabolism qualities [5], has been thought to be the basis to its possible health benefits. For this reason, it has been proposed that coconut oil not be considered in the same context as other saturated fats and has been termed by some proponents as a “healthy fat” [6, 7].

There have been few case reports or research into the effects of coconut oil on energy metabolism, specifically glycemic changes. We present a case of a 66-year-old man with a history of type 2 diabetes mellitus managed with insulin who developed recurrent hypoglycemia and required reduction in insulin therapy quickly after consuming coconut oil supplementation. We review the current literature on coconut oil's effect on energy metabolism and glucose homeostasis and propose future areas of research into these properties of coconut oil.

## 2. Case

A 66-year-old male presented to the endocrine clinic for consultation regarding new onset hypoglycemia. He was diagnosed with type 2 diabetes mellitus at age 42 and was initially managed with metformin. Subsequently, insulin was added due to worsening glycemic control. His insulin regimen, until recently, consisted of NPH insulin 20 units subcutaneously twice daily and regular insulin 15 units before each meal. A few weeks prior to presentation to the endocrine clinic, he started taking coconut oil supplements because he heard about some health benefits. Specifically, he started taking a dose of 1000 mg daily. Within 1-2 days of starting the supplement, he began experiencing hypoglycemia. He denied any changes in his appetite or caloric intake. There were no other factors that would have explained his onset of hypoglycemia. Specifically, his weight, which was 253 lbs at baseline (for a body mass index of 33.5), had not changed. He did not start taking any other new medications or supplements. Based on advice from his primary care provider, he reduced his insulin doses: initially, NPH down to 15 units twice daily and then to 10 units twice daily. He then started avoiding some of the regular insulin doses. When he took regular insulin, he would have hypoglycemia at bedtime.

During his visit to the endocrine clinic, he reported a stable weight. He was experiencing hypoglycemia (defined as blood glucose below 70 mg/dL [8]) about once weekly. These episodes tended to occur after he had supper, close to bedtime. His lowest glucose reading was 50 mg/dL in the past 2 weeks. His hemoglobin A1c, a long-term measure of glycemic control, was 6.2%. This was lower than his previous level of 6.4% checked 3 months prior to starting coconut oil, despite cutting the dose of his long-acting insulin in half and occasionally skipping his meal-time insulin. His lipid levels prior to coconut oil intake were as follows: total cholesterol of 116 mg/dL, LDL of 36 mg/dL, HDL of 34 mg/dL, and triglycerides of 232 mg/dL. He was taking a statin medication before and continued to take it after initiating coconut oil. By the time he was seen in the endocrine clinic, a lipid panel was not repeated, due to short-time duration since being on the new supplement. The patient was intending to raise his coconut oil dose to 2000 mg per day and had questions regarding the effects of coconut oil on his blood glucose and implications of taking coconut oil supplementation on his diabetes management. This prompted a review of the literature as detailed in the remainder of this report.

## 3. Discussion

Research into the effects of coconut oil has increased over the past few decades. It has been proposed to have anti-inflammatory, antioxidant, enhanced metabolism, as well as antidiabetic properties. Coconut oil has been implicated in improving the quality of life in cancer treatments, reducing inflammation markers, reducing toxic side effects of chemotherapy, and even improving bone mineral density in animal studies [9–14]. The metabolism of coconut oil has been heavily researched. Coconut oil is high in saturated fatty acids, with more than 60% consisting of medium-chain fatty acids (MCFAs), a large percentage of which is lauric acid (C 12 : 0). Its medium-chain fatty acid property has been thought to be the source of its health benefits as MCFA have been shown to improve metabolism and positively affect cardiometabolic risk factors [5]. The metabolism of MCFA differs from other fatty acids as they are hydrolyzed and absorbed faster than other fats. They do not undergo degradation and re-esterification processes and can be directly used in the body to produce energy. This is thought to result in faster gastric emptying, thus leading to less stimulation of gut hormones [6, 15]. After their absorption, MFCAs are transported directly into the liver and undergo *β*-oxidation. Compared with long-chain fatty acids (LCFAs), MCFAs do not require chylomicron transport via the lymphatic system to reach their target tissues, thereby favoring hepatic metabolism and mitochondrial oxidation [2, 16]. This results in preferential metabolism of MCFAs over LCFAs, and different animal and human trials have proposed this property to positively effect satiety, weight loss, energy expenditure, and glucose homeostasis [5, 17].

One of the most heavily researched aspects of coconut oil is its effect on lipid metabolism and cardiovascular risk reduction. Epidemiologic studies have found that population groups that consume coconut as part of their traditional diets had higher HDL and lower rates of cardiovascular disease [2]. It is important to note that the type of coconut these populations consume is not the same as what is in a typical Western diet and in processed coconut products. Results of studies on effects of coconut oil on cholesterol metabolism have been mixed, with most studies favoring an increase in HDL, LDL cholesterol and triglyceride levels [18–20]. The proposed mechanisms are thought to be due to effects of lauric acid, which acts as a substrate in the liver for the production of ApoA-I and ApoB production. A second mechanism is through a decrease in HDL cholesterol and ApoA-I catabolism and increased reverse cholesterol transport due to increased size of the HDL cholesterol molecule [9]. One randomized control trial found that HDL cholesterol levels increased on average by 3.1 mg/dL with coconut oil supplementation [19]. Some evidence (though inconsistent) points to butter raising LDL cholesterol more than coconut oil. When coconut oil replaces carbohydrates, a slight reduction in LDL-to-HDL cholesterol ratio has been reported. However, when coconut oil replaces monounsaturated or polyunsaturated oils, LDL is raised. Since cardiovascular risk is more correlated with raising LDL than raising HDL and because coconut oil increases LDL cholesterol, a cause of CVD, the American Heart Association Presidential advisory panel in 2017 recommended against the use of coconut oil [4]. This recommendation is further supported by 2 recent systematic reviews and meta-analyses on the effects of coconut oil on cardiovascular risk factors. One review included 16 trials and showed that compared with nontropical vegetable oils, coconut oil consumption increased total cholesterol, LDL cholesterol, and HDL cholesterol and did not significantly change C-reactive protein, fasting glucose concentrations, or measures of body adiposity. The findings prompted the authors to express concern about increased consumption of the saturated fat, coconut oil, in the population [21]. Another review, which included 12 trials, showed similar findings on cholesterol profile when compared with other plant oils. However, in this review, a comparison with animal oils found that coconut oil reduced LDL cholesterol. Despite the HDL cholesterol-raising effect, the authors were cautious to not recommend replacing a significant amount of unsaturated plant oil with coconut oil [22]. The latter review did not evaluate effects on glycemia.

The patient in this case report developed recurrent hypoglycemia and had decreased insulin requirements after the addition of coconut oil supplementation to his diet. We hypothesize that the metabolic and anti-inflammatory properties of coconut oil contributed to the patient's decreased insulin requirements. This is the first known case report of coconut oil supplementation in a diabetic patient on insulin resulting in hypoglycemia. There have been multiple studies into the effects of coconut oil on glucose homeostasis in animal models. In a study on streptozocin-induced diabetic rats fed various types of fats, the coconut oil-fed rats exhibited the best (lowest rise in glucose) response to an oral glucose tolerance test (compared with palm oil and groundnut oil). These same rats also exhibited lower levels of total cholesterol and non-HDL cholesterol. It was hypothesized that the mechanism of improved insulin sensitivity was related to the triglyceride levels, which were reduced in the coconut oil-fed rats [23]. This may or may not be generalizable to human populations where triglyceride levels have been found to rise after coconut oil supplementation. A more recent study on alloxan-induced diabetic rats showed that virgin coconut oil intake resulted in reduction in food intake and body weight gain, as well as improvement in microbiota profile (increase in certain probiotic bacteria) in diabetic rats [24]. A study on C57/BL6 male mice fed a diet moderately high in fat from coconut oil + soybean oil vs. coconut oil only or fructose only showed that addition of soybean oil was most detrimental to metabolic outcomes such as diabetes, insulin resistance, and fatty liver [25]. Another study on a similar mouse model was mainly conducted to evaluate the effect of coconut oil on cardiomyopathy (vs. standard chow diet). Though it did show an aggravation of cardiomyopathy from coconut oil consumption, specific effects on insulin resistance were evaluated and found to be neutral [26]. In an attempt to better mimic a human diet-induced obesity model, one study in female Ossabaw minipigs fed a high fat diet rich in hydrogenated fats, fructose, and coconut oil surprisingly showed that despite developing obesity, those pigs manifested similar glucose concentrations and lower systemic inflammatory cytokine levels compared with controls, as well as a shift of their urogenital microbiota towards a more anti-inflammatory phenotype. It was hypothesized that coconut oil in their diet conferred those benefits [27]. Finally, it is worth noting that the various chemical forms of coconut oil may not behave similarly. A study on the effect of thermally oxidized coconut oil (the kind that would naturally occur from cooking at high temperatures and not what is present in virgin coconut oil supplements) on glucose metabolism in rats showed negative effects. Specifically, those rats were fed a high-fructose diet and developed insulin resistance, hyperglycemia, and systemic inflammation. Those effects were all exacerbated, or made worse, in the group supplemented with thermally oxidized coconut oil [28]. All of these studies combined show that the data on the effect of coconut oil on glucose homeostasis in animals have shown some favorable, but overall mixed results.

As far as human studies on effects of coconut oil intake on glucose homeostasis, an older study conducted in 1992 by Eckel et al. showed improved insulin mediated glucose metabolism after medium-chain triglyceride (MCT) supplementation for four days (vs. isocaloric long-chain triglyceride supplementation). This effect was seen in subjects with and without type 2 diabetes, and in diabetics, it was independent of severity of diabetes or intake of sulfonylureas. Using the euglycemic clamp technique following an MCT supplemented diet, the amount of glucose needed to maintain euglycemia during an intravenous insulin infusion was increased in diabetic subjects by 30%, in nondiabetic subjects with hypertriglyceridemia by 30%, and in normotriglyceridemic subjects by 17%, all indicating higher insulin sensitivity [29]. More recently, a randomized trial on 75 adult obese women all following a hypocaloric lifestyle modification program divided the women into 4 groups of vegetable oil supplementation: coconut, safflower, chia, and soybean. Compared to baseline, all groups saw a significant (*p* < 0.001) reduction in weight, LDL cholesterol, triglycerides, blood glucose, and hemoglobin A1c and an increase in HDL cholesterol. Addition of 6 grams of coconut oil supplementation to the diet resulted in the largest decrease amongst the 4 groups in average blood glucose (−25 mg/dL vs. baseline) and in glycated hemoglobin A1c (−0.86% vs baseline). The coconut oil group also saw the greatest reduction in weight (about 8% reduction vs. baseline), with a concomitant reduction in body mass index, waist-to-height ratio, and increase in % lean mass. The coconut oil consumed about 613 less Kcal/day at the end of the study compared to baseline, which was significantly lower than the calorie reduction in the other three groups. The authors hypothesized that this preferential weight loss effect may have been related to increased satiety effects of coconut oil. Most of the calorie reduction was from reduced carbohydrate intake. The glycemic effects were also hypothesized to be related to phenolic compounds in coconut oil, discussed in the next paragraph of this report, although these compounds were not directly measured. The chia seed oil saw the greatest improvement in lipid parameters [17]. Finally, a randomized crossover controlled study on 15 adult women with excess body fat (defined as far comprising >32% of body composition) evaluated acute effects of virgin coconut oil (VCO) administration on energy metabolism, cardiometabolic risk markers, and appetitive responses. Subjects were fed a breakfast supplemented with 25 mL of MCFA in the form of VCO (vs. control: olive oil), and postprandial data were obtained for up to 240 minutes. The VCP group did not exhibit any significant differences in postprandial glucose response compared to placebo. Also, notable was that the VCO group has less satiety response than placebo. There were no differential effects on lipid levels. It was hypothesized by the authors that differences in fatty acid composition, and hence structure, of VCO (compared to other synthetic forms of coconut oil) may explain some of these negative findings. This prompted the authors to caution against prescribing this form of coconut oil as a supplement, given no clear benefit in this acute study [15]. The above human studies show that data remain mixed on effects of coconut oil supplementation on glucose homeostasis in humans. This may be related to varying forms of coconut oil supplementation, as well as confounding of weight loss from calorie restriction. One important observation in the case report presented in this current paper is that the hypoglycemic effects were seen after 2 days of ingestion of 1 g of coconut oil supplements. The human studies cited above had durations that varied from 240 minutes postprandial (which showed a negative result) to 4 days (using 40 grams of MCT oil/1000 calories per day) to 8 weeks (using 6 grams/day of coconut oil supplementation). This shows that it is difficult at this point, based on the data reported in the literature, to predict or validate whether ingestion of 1 g per day for 2 days is sufficient enough to show a response. More studies are needed before a consensus is reached on the glycemic effects.

When it comes to the proposed mechanisms to any likely benefit on glycemia, coconut oil's high concentration of polyphenolic compounds has been proposed to increase the efficiency of glucose metabolism [1]. Coconut oil is known to contain phenolic antioxidants such as caffeic acid, ferulic acid, syringic acid, catechin, and epigallocatechin. Phenolic compounds have been shown to have antidiabetic and insulin-sensitizing effects, as well and anti-inflammatory and immune modulatory priorities [17, 30]. Phenolic compounds have been implicated in inhibiting the COX pathway, downregulating TNF alpha and cytokine production, and inactivating peroxisome proliferator-activated receptor gamma (PPAR*γ*) [30]. Given these properties, it has been proposed that coconut oil ingestion results in less demand for enzyme production of the pancreas, thus improving insulin secretion and utilization of blood glucose [31]. One specific example is ferulic acid, which has been shown to contain high levels of phenolic acids, and when added to the diet in type 2 diabetes mice models, resulted in reduction in serum glucose, increase in serum insulin, and increased hepatic glycogen production and glucokinase activity [32]. Coconut kernel protein has also been shown to have antidiabetic properties, hypothesized to improve pancreatic beta-cell regeneration by means of arginine [16, 33]. One challenge remains and that is to determine the correlation between coconut oil doses, phenolic content, and clinically meaningful antioxidant activity. Rigorous studies using various extraction methods have shown that the antioxidant properties of coconut oil correlate with phenolic compound content, and that both of those vary with the method of extraction used. Virgin coconut oil (VCO), especially when produced by the chilling method, tends to have the highest phenolic acid content (as measured by gallic acid equivalents) and antioxidant activity (as measured by scavenging activity of the stable 1,1-diphenyl-2-picrylhydrazyl (DPPH) free radical, b-carotene-linoleate bleaching activity, and reducing power) [34, 35]. In one study, the total phenolic content and DPPH radical-scavenging activity of VCO was found to be in the range of 1.16–12.54 mg gallic acid equivalents (GAE)/g and 7.49–104.52 mg/ml [35]. However, the correlation of this data with the amounts usually present in supplements (1000 mg per supplement) or in diets rich in coconut oil, and their clinical relevance, i.e., the dose needed to give a clinical effect, has not been published. More controlled experiments need to be done to answer these questions.

## 4. Back to the Patient

The patient presented in this case report was advised to stop the prandial insulin due to evidence of postprandial hypoglycemia. He was started on a GLP-1 agonist medication to help maintain good glycemic control as the prandial insulin was withdrawn. He was given dietary advice specific to diabetes management and weight loss. He was advised to continue taking the statin medication for cardiovascular prevention given his age and history of diabetes. He wished to continue the coconut oil supplements knowing that the evidence for their use is not consistent. The next steps in his management will include attempting to reduce his long-acting insulin (NPH) if enough weight is lost. At the time of completion of this case report, the patient had not returned for a follow-up visit to the endocrine clinic.

## 5. Conclusion

This is the first known case report of coconut oil supplementation in a diabetic patient on insulin resulting in hypoglycemia. Summary of the literature shows that coconut oil supplementation may have an effect on glycemic control, possibly through phenolic compounds mediating anti-inflammatory effects. This effect is inconsistent throughout the studies reviewed, likely due to variations in types of coconut oil supplementation and scarcity of animal mechanistic studies and human clinical trials. One of the limitations of this case report is that it was not a controlled experimental trial, and patient report was the primary source of data; thus, we cannot conclude with certainty that it was coconut oil ingestion that resulted in the reported hypoglycemia. This case report is clinically relevant because it highlights the importance of checking supplement history in patients in general and specifically in patients with diabetes. Patients with diabetes are at increased risk of cardiovascular complications, and this is especially important since coconut oil is a saturated fat that has been shown to increase LDL cholesterol. Therefore, even if the evidence points to some improvement in glycemic control, further outcomes research is required in humans before coconut oil intake is advised and popularized, to the general population as well as to patients with diabetes in particular.

## Data Availability

The data used to support the findings of this study are avilable from the corresponding author upon request.

## Abbreviations

BG: Blood glucose
MCFA: Medium-chain fatty acids
SFA: Saturated fatty acids
VCO: Virgin coconut oil.
